# Supplementary material for: A high-resolution mRNA expression time course of embryonic development in zebrafish
Source: eLife. 2017 Nov 16;6:e30860. doi: 10.7554/eLife.30860 (PMC5690287; doi:10.7554/eLife.30860)
Supplement: Supplementary file 6. [file elife-30860-supp6.zip › biolayout-clusters-files/Cluster191-genes.html]

Cluster191


# Cluster191: Genes

| Ensembl ID | Gene Name | Chr | Start | End | Biotype |
| --- | --- | --- | --- | --- | --- |
| ENSDARG00000102769 | CDH18 (1 of many) | 12 | 41814596 | 41965517 | protein\_coding |
| ENSDARG00000102125 | IQSEC2 (1 of many) | 8 | 22597066 | 22718712 | protein\_coding |
| ENSDARG00000062956 | dagla | 7 | 13130132 | 13234505 | protein\_coding |
| ENSDARG00000009626 | nrbp2b | 24 | 33311911 | 33361719 | protein\_coding |
| ENSDARG00000010816 | slc7a14a | 2 | 26212770 | 26335150 | protein\_coding |
| ENSDARG00000079931 | snx19b | 15 | 17505712 | 17553704 | protein\_coding |
| ENSDARG00000044514 | st6gal1 | 21 | 31113743 | 31147340 | protein\_coding |
